# Sulfur Vacancies Limit the Open-Circuit Voltage of Sb_2_S_3_ Solar Cells

**DOI:** 10.1021/acsenergylett.4c02722

**Published:** 2024-12-16

**Authors:** Xinwei Wang, Seán R. Kavanagh, Aron Walsh

**Affiliations:** † Department of Materials, Imperial College London, Exhibition Road, London SW7 2AZ, U.K.; ‡ Center for the Environment, Harvard University, 29 Oxford St, Cambridge, Massachusetts 02138, United States; ¶ Department of Physics, Ewha Womans University, 52 Ewhayeodae-gil, Seodaemun-gu, Seoul 03760, South Korea

## Abstract

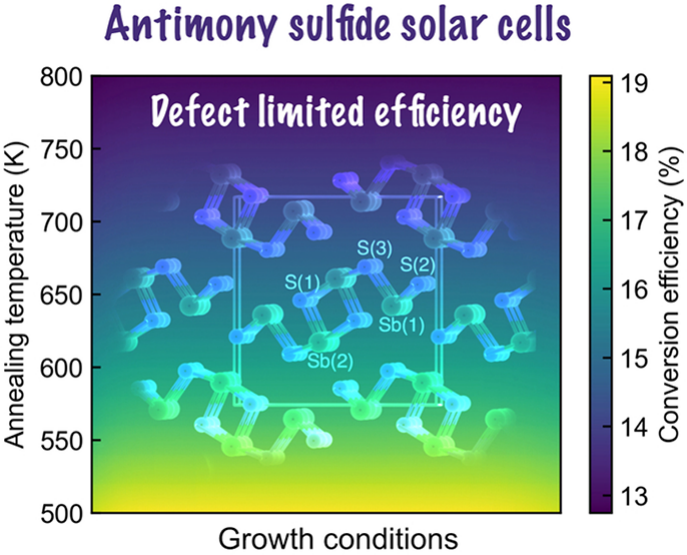

Antimony sulfide (Sb_2_S_3_) is a promising candidate as an absorber layer for single-junction solar cells and the top subcell in tandem solar cells. However, the power conversion efficiency of Sb_2_S_3_-based solar cells has remained stagnant over the past decade, largely due to trap-assisted nonradiative recombination. Here we assess the trap-limited conversion efficiency of Sb_2_S_3_ by investigating nonradiative carrier capture rates for intrinsic point defects using first-principles calculations and Sah–Shockley statistics. Our results show that sulfur vacancies act as effective recombination centers, limiting the maximum light-to-electricity efficiency of Sb_2_S_3_ to 16%. The equilibrium concentrations of sulfur vacancies remain relatively high, regardless of growth conditions, indicating the intrinsic limitations imposed by these vacancies on the performance of Sb_2_S_3_.

Antimony sulfide (Sb_2_S_3_) has attracted great research interest as an emerging light-absorbing material for next-generation photovoltaic (PV) devices, driven by its earth-abundant and environmentally friendly constituents, as well as its attractive optical and electronic properties.^1^ Sb_2_S_3_ has a high optical absorption coefficient (>1 × 10^4^ cm^–1^ in the visible region),^2^ decent carrier mobility,^3,4^ and excellent thermal and chemical stability.^5^ Its band gap of 1.7–1.8 eV^1^ is well-aligned with the spectra of indoor light sources and is ideal for the top subcell in tandem solar cells. Additionally, its relatively low melting point (550 °C^1^) facilitates the growth of high-quality Sb_2_S_3_ crystalline films at moderate temperatures. Despite these advantages, the power conversion efficiencies (PCEs) of single-junction Sb_2_S_3_ solar cells remain low. The highest recorded efficiencies are 8.3% for the planar geometry type^6^ and 7.5% for the sensitized type,^7^ far below the thermodynamic limit of ∼30% for a material with this band gap.^8^

The main challenge impeding further efficiency improvements in Sb_2_S_3_ solar cells is the significant open-circuit voltage (*V*_OC_) deficit. Despite various device architectures and fabrication strategies,^5^ the *V*_OC_ deficit for the most efficient Sb_2_S_3_ devices remains greater than 0.9 V,^6^ indicating a high electron–hole recombination rate. The detailed balance principle^8^ predicts a minimum *V*_OC_ deficit (defined as *E*_g_/*q* – *V*_OC_^SQ^) of ∼0.27 V due to unavoidable band-to-band radiative recombination at 300 K for a material with a band gap of 1.7 eV.^9^ Besides band-to-band recombination, electron and hole capture processes can occur nonradiatively through multiple-phonon emission (either via defect-mediated processes or the Auger–Meitner effect^10,11^) or radiatively via defect-mediated pathways that involve the emission of photons. For defect-mediated capture processes, radiative capture cross sections are typically on the order of 10^–5^–10^–4^ Å^2^, which are significantly smaller than the 10^–2^–10^4^ Å^2^ range for nonradiative processes. The Auger–Meitner process^11^ becomes relevant only in systems with exceptionally high defect and carrier concentrations (usually >10^17^ cm^–3^).^10^ Consequently, defect-mediated nonradiative recombination (Shockley–Read–Hall (SRH) recombination) is widely recognized as the dominant loss mechanism in Sb_2_S_3_ solar cells.^12^

There is ongoing debate regarding whether SRH recombination predominantly occurs at the surface/interface or within the bulk of Sb_2_S_3_^13^ and whether vacancies or antisites are the most detrimental defects.^14^ There have also been predictions around multicarrier trapping.^15^ Identifying the specific type of defect can be challenging when relying solely on experimental methods, making complementary theoretical simulations essential. Previous studies on defect identification in Sb_2_S_3_ have primarily focused on comparing energy levels and defect types (acceptor vs donor). However, due to the low crystal symmetry of Sb_2_S_3_, there are multiple types of defects with numerous trap states distributed across the band gap. Furthermore, not all deep-level traps are active recombination centers capable of rapid carrier capture. A more comprehensive understanding of charge carrier recombination kinetics through defects is therefore essential. Traditional SRH theory^16,17^ is commonly used to calculate recombination rates via single-level defects. Nevertheless, many studies in this field continue to rely on the SRH theory with additional approximations for defects with multiple energy levels. Some researchers neglect the carrier re-emission processes and derive effective total carrier capture coefficients,^18^ while others treat each defect level independently and sum the SRH recombination rates for individual single-level defects.^19,20^ These approximations, however, can lead to significant errors in systems with correlated defect transitions or negative correlation energies,^21,22^ such as antimony chalcogenides.^23^ For such systems, Sah–Shockley statistics,^24^ which account for multiple defect levels, provide more accurate predictions.

In this work, we have performed systematic first-principles calculations to investigate intrinsic point defects in Sb_2_S_3_ using a global structure searching approach ShakeNBreak.^23,25,26^ We further assessed nonradiative carrier recombination via these defects using Sah–Shockley statistics.^24^ By accounting for both band-to-band radiative and trap-mediated nonradiative recombination, we predict the upper limit of power conversion efficiency (PCE) in Sb_2_S_3_. Our results reveal that sulfur vacancies are the most detrimental defects, contributing significantly to *V*_OC_ loss due to their consistently high equilibrium concentrations under a range of growth conditions.

Sb_2_S_3_ forms an orthorhombic crystal structure and belongs to the *Pnma* space group^29^ (as shown in Figure 1a). The structure consists of quasi-one-dimensional (1D) [Sb_4_S_6_]_*n*_ ribbons along the [100] direction, which are linked together by weak interactions.^30^ The low crystal symmetry of the structure results in distinct coordination environments for each Sb and S element within the unit cell, leading to two inequivalent Sb sites and three inequivalent S sites, all of which were considered in our calculations.

**Figure 1:**
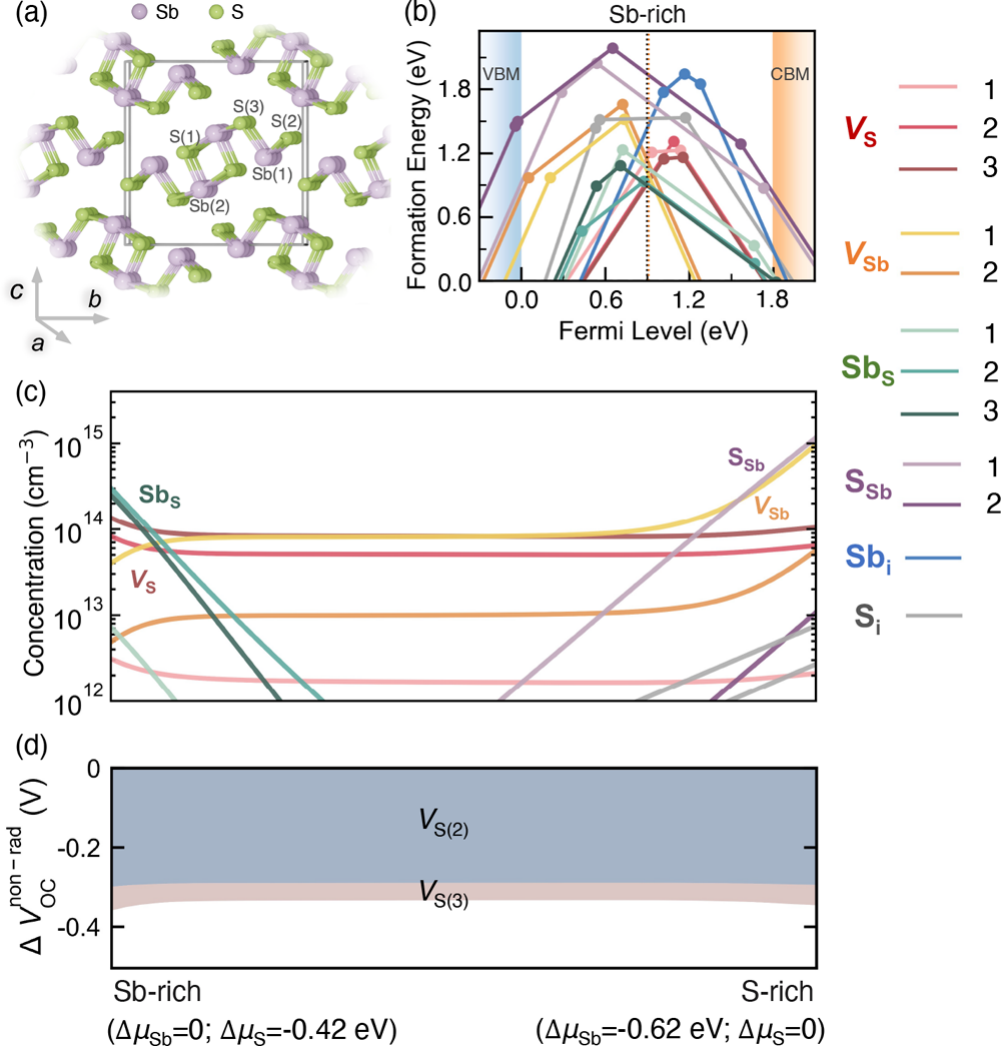
(a) Ground-state crystal structure (*Pnma* space group) of Sb_2_S_3_. The crystallographic unit cell is represented by a cuboid. Inequivalent sites are denoted by the atom labels enclosed in parentheses. (b) Calculated formation energies of intrinsic point defects in Sb_2_S_3_ under Sb-rich growth conditions using doped(27) and ShakeNBreak.^25^ The slopes of solid lines represent charge states, and the filled circles indicate thermodynamic transition levels. The valence band maximum (VBM) is set to 0 eV, and the conduction band minimum (CBM) is obtained from the calculated fundamental (indirect) band gap of 1.79 eV by the HSE06 functional. Vertical dashed lines in orange and black, respectively, indicate self-consistent Fermi levels at 603 K^6,28^ and at 300 K in Sb_2_S_3_ crystals grown at 603 K.^6,28^ The numbers in the legend correspond to different inequivalent sites; for interstitials, only the lowest energy states at each charge state are shown. (c) Equilibrium defect concentration at 300 K in Sb_2_S_3_ crystals grown at 603 K^6,28^ as a function of the growth condition. (d) *V*_OC_ deficit contributed by nonradiative recombination (Δ) in Sb_2_S_3_ as a function of the growth condition, decomposed into individual defect contributions. Δ is defined as the difference between the values of *V*_OC_ and . Defect species with Δ 0.05 V are not shown. Film thickness is assumed to be 400 nm.^6,28^

## Equilibrium Bulk Defects

All intrinsic point defects, including vacancies, antisites, and interstitials, were systematically investigated by first-principles calculations. Details of defect generation and optimization are provided in Methods. The formation of defects under various experimental growth conditions can be described by its dependence on the chemical potentials of the constituent elements (see Section S2.1 for details). The defect formation energy (DFE) diagram in Figure 1b plots the thermodynamically stable charge states as a function of the Fermi level (E_F_) within the band gap under Sb-rich conditions as an example of typical systhesis conditions. Other growth conditions are shown in Figure S2. Similar to previous studies on Sb_2_Se_3_,^31^ all intrinsic point defects in Sb_2_S_3_ exhibit amphoteric behavior, with stable positive and negative charge states depending on the position of E_F_. This suggests strong charge compensation, which reduces carrier density and leads to poor electrical conductivity, consistent with other reports.^32^ All point defects with low formation energies have deep thermodynamic transition levels (TLs), making it challenging to identify detrimental defects solely on the basis of their deep-level characteristics.

The equilibrium defect concentration as a function of chemical potential is further calculated under the constraint of charge neutrality.^33^ As illustrated in Figure 1c, under Sb-rich conditions, Sb_S_ and *V*_S_ are dominant defects with high concentrations (>10^14^ cm^–3^). As the sulfur chemical potential (μ_S_) increases, the density of Sb_S_ decreases significantly, while that of S_Sb_ rises sharply. In contrast, the variation in the vacancy concentration with μ_S_ is less pronounced. *V*_S(2)_ and *V*_S(3)_ maintain consistently high concentrations across various growth conditions. While the concentration of *V*_Sb_ initially increases slowly with μ_S_, it then increases dramatically as the system approaches S-rich conditions, ultimately reaching a high concentration under S-rich conditions. The insensitivity of defect concentrations to growth conditions can be explained by defect-correlations,^34^ with Schottky-type disorder between *V*_S_ and *V*_Sb_ leading to charge compensation across most of the chemical potential range. Specifically, as μ_S_ increases from S-poor to S-rich conditions, the formation energies of *V*_S_ and Sb_S_ shift upward, while those of *V*_Sb_ and S_Sb_ shift downward, leading to a reduction in the self-consistent Fermi level (Figure S2). Due to the positive (negative) charge states of *V*_S_ (*V*_Sb_), the decreasing Fermi level lowers (raises) their formation energies (Δ*E*_*D*,*q*_^*f*^ ∝ *qE*_*F*_), counterbalancing the effect of chemical potential and resulting in relatively stable equilibrium concentrations of *V*_S_ and *V*_Sb_. All interstitials are found to have low concentrations, which agrees with the experimental observation that interstitials have a negligible impact on carrier lifetime.^35^

We note that previous first-principles studies on Sb_2_S_3_^34,36,37^ commonly reported sulfur vacancies as donors and antimony vacancies as acceptors, rather than amphoteric defects. This discrepancy likely stems from the absence of global structure-searching methods and the limited exploration of charge states in these earlier studies. Therefore, our findings emphasize the importance of using a global structure search for accurate defect predictions in chalcogenide semiconductors.

## Carrier Capture under Steady-State Illumination

The defect-mediated carrier capture processes via multiphonon emission and corresponding recombination kinetics in Sb_2_S_3_ were then investigated. The complete pathways for electron and hole capture by defects with high carrier concentrations can be found in Figure S3. The maximum achievable conversion efficiency is further predicted to quantify the impact of trap-assisted electron–hole recombination on the performance of Sb_2_S_3_ solar cells. The predicted open-circuit voltage *V*_OC_ deficit due to radiative recombination is 0.10 V. Nonradiative recombination contributes significantly to the total *V*_OC_ deficit, with values of 0.45 and 0.44 V under Sb-rich and S-rich conditions, respectively (Figure S4a). Further analysis of the *V*_OC_ loss due to nonradiative recombination (Δ) shows that the highest loss of 0.36 V occurs under Sb-rich conditions, with losses ranging from 0.33 to 0.36 V across different growth conditions (Figure 1d). To identify the most detrimental defect species, the contributions to Δ are divided by individual defect type. As shown in Figure 1d, the conversion efficiency of Sb_2_S_3_ is primarily limited by sulfur vacancies, whereas antimony vacancies, antisites, and interstitials have negligible impact on nonradiative recombination. Among the sulfur vacancies, *V*_S(2)_ and *V*_S(3)_ are found to be the most harmful, owing to their deep transition levels, high concentrations and large carrier capture coefficients for both electrons and holes, while the relatively low concentration of *V*_S(1)_ (∼10^12^ cm^–3^) under various growth conditions leads to its negligible impacts on the performance. Minimizing *V*_S(2)_ and *V*_S(3)_ are thus crucial for improving the PCE of Sb_2_S_3_ solar cells.

As previously discussed, the primary defect species contributing to the largest *V*_OC_ loss is predicted to be *V*_S(2)_ (Figure 1d), an amphoteric defect with multiple accessible charge states: 0, ±1, and ±2. The overall recombination rate of electrons and holes via such a multivalent defect is determined by competing transitions between different charge states, rather than by the sum of individual transitions. Consequently, the commonly used SRH statistics, which address only the transition between two charge states, may be inadequate, so we also implement a treatment based on Sah–Shockley statistics which includes transitions between multiple charge states.^24^

**Figure 2:**
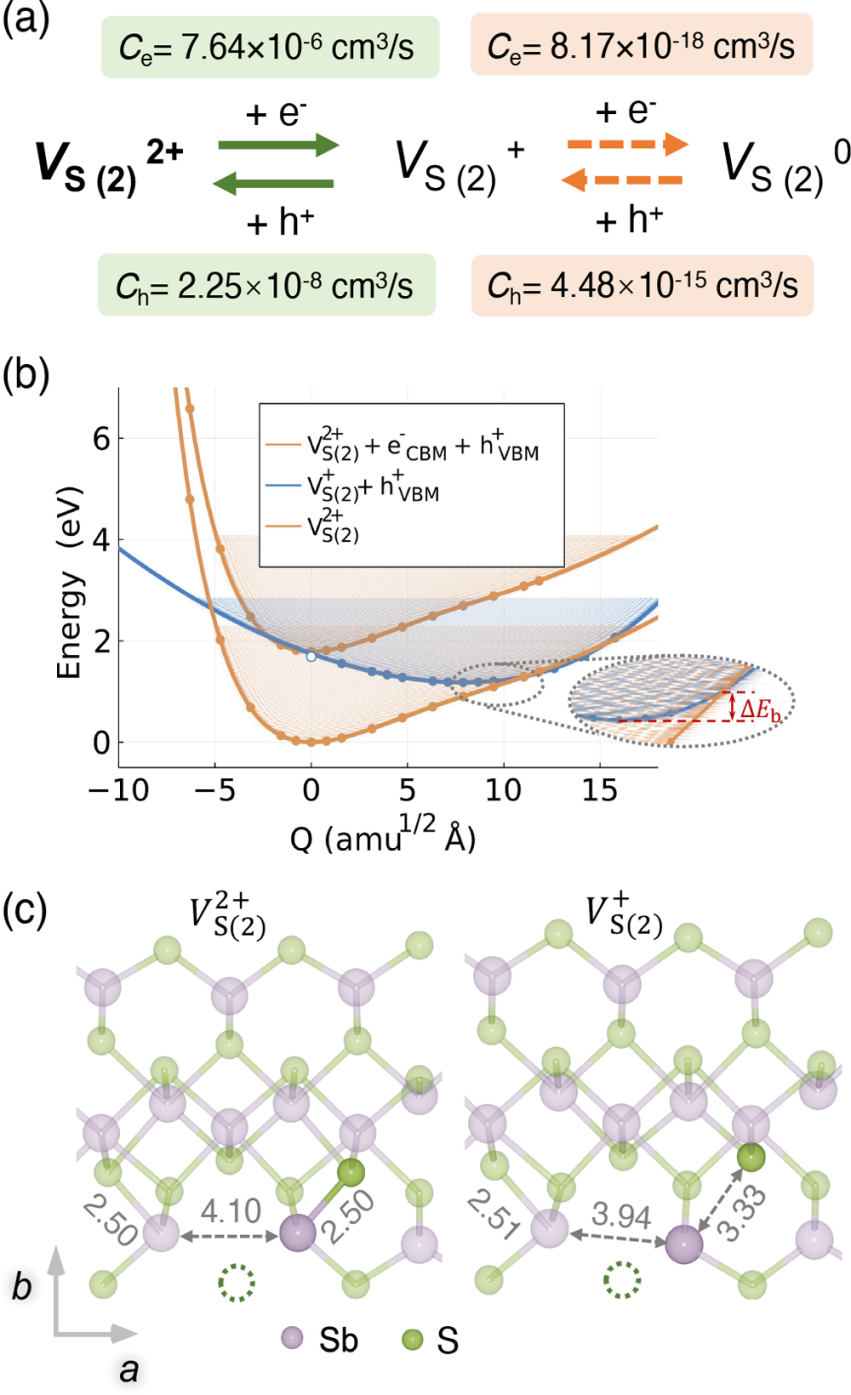
(a) Pathways for electron and hole capture by *V*_S(2)_. *C*_*e*_ and *C*_*h*_ are electron and hole capture coefficients, respectively, calculated using CarrierCapture.^38^ Green and orange indicate rapid and slow capture processes, respectively. (b) One-dimensional (1D) configuration coordinate diagram for charge transitions between *V*_S(2)_^2+^ and *V*_S(2)_^+^. Solid circles are data points obtained by DFT calculations and used for fitting, while hollow circles are discarded for fitting due to charge delocalization. Solid lines represent the best fits to the data. The transition barrier Δ*E*_b_ for one capture process corresponds to the energy difference between the minimum of the initial state and the crossing point with the final state. (c) Defect configurations of *V*_S(2)_^2+^ and *V*_S(2)_^+^. The bond lengths in Å are labeled, and the vacant S site is denoted by a dotted circle.

For carrier capture transitions involving *V*_S(2)_, the *V*_S(2)_^2+^ state is considered as the starting point, since it has the highest equilibrium concentration under various growth conditions (Figure 1b and Figure S1). The detailed carrier capture transition pathways associated with *V*_S(2)_ are illustrated in Figure 2a. In nonradiative carrier recombination processes, *V*_S(2)_^2+^ captures an electron from the conduction band minimum (CBM), followed by hole capture by *V*_S(2)_^+^ from the valence band maximum (VBM). These capture processes can be described by using a configuration coordinate (cc) diagram. As shown in Figure 2b, the potential energy surfaces (PESs) of *V*_S(2)_^2+^ and *V*_S(2)_^+^ are plotted as a function of 1D generalized coordinate *Q*, which represents atomic displacement (Figure 2b). The coordinate *Q* is generated by linearly interpolating between the ground-state configurations of *V*_S(2)_^2+^ and *V*_S(2)_^+^, and it corresponds to the vibrations most strongly coupled to the structural distortion during the transition. The validity of this 1D approximation is supported by the linear fit of the wave function overlap ⟨ψ_*i*_|ψ_*f*_⟩ as a function of *Q* (shown in Figure S5).

The lifetime for carrier capture processes is inversely proportional to the product of the defect concentration and carrier capture coefficients. The latter suggests how fast electrons or holes are captured by the defect, which can be calculated quantum-mechanically using Fermi’s golden rule^10^ from the cc diagram. The detailed procedure to calculate capture coefficients was outlined in our previous work.^31^Table 1 summarizes the carrier capture coefficients and cross sections at room temperature, along with key parameters used in the calculations performed within CarrierCapture.^38^ The large electron–phonon matrix element *W*_*if*_ shows a strong promoting character of the configuration coordinate. The mass-weighted displacement Δ*Q* quantifies the structural difference between the two defect charge states involved in the capture process. For the transition between *V*_S(2)_^2+^ and *V*_S(2)_^+^, the main contribution to Δ*Q* of 7.88 amu^1/2^ Å arises from the shortening (lengthening) of a Sb–S bond adjacent to *V*_S(2)_ during the electron (hole) capture process (Figure 2c). The PESs were generated by interpolating between the equilibrium structures of *V*_S(2)_^2+^ and *V*_S(2)_^+^ using single-point functional density theory (DFT) calculations (Figure 2b). During the nonradiative electron capture process by *V*_S(2)_^2+^, the initial (i.e., excited) state is represented by the uppermost orange curve, while the final (i.e., ground) state corresponds to the blue curve. The two PESs intersect at Δ*E*_b_ = 5 meV above the minimum of the excited state. This small Δ*E*_b_, combined with a large phonon overlap, results in a large electron capture coefficient (*C*_*e*_) of 7.64 × 10^–6^ cm^3^s^–1^ at room temperature. For hole capture by *V*_S(2)_^+^, the initial and final states are represented by the blue and bottom-most orange curves, respectively. The weak Coulomb repulsion between holes and *V*_S(2)_^+^, the reduced pathway degeneracy *g* and a larger Δ*E*_b_ of 121 meV (Table 1), contribute to a smaller hole capture coefficient (*C*_*h*_) of 2.25 × 10^–8^ cm^3^ s^–1^ at room temperature. Subsequent electron capture by *V*_S(2)_^+^ or hole capture by *V*_S(2)_^0^ proceeds much more slowly, with capture coefficients <1 × 10^–14^ cm^3^ s^–1^ (Figure 2c). Therefore, the *V*_S(2)_^2+^ ⇄ *V*_S(2)_^+^ recombination cycle is efficient, making the overall electron–hole recombination process at *V*_S(2)_ primarily limited by the hole capture process .

**Table 1:** Key Parameters Used to Calculate the Carrier Capture Coefficients in the Transition of *V*_S(2)_^2+^ ↔ *V*_S(2)_^+^^a^

Species	Δ*Q*	Capture process	Δ*E*_b_	*g*	*W*_*if*_	*s*(*T*)*f*	*C*	σ
*V*_S_	7.88	Electron	5	4	1.65 × 10^–2^	5.34	7.64 × 10^–6^	4.14 × 10^–13^
		Hole	121	1	3.22 × 10^–2^	0.36	2.25 × 10^–8^	1.54 × 10^–15^

a Mass-weighted distortion Δ*Q* (amu^1/2^ Å), energy barrier Δ*E*_b_ (meV), degeneracy factor *g* of the final state, electron-phonon coupling matrix element *W*_*if*_ and scaling factor *s*(*T*)*f* at 300 K, along with calculated capture coefficient *C* (cm^3^ s^–1^) and cross-section σ (cm^2^) at 300 K.

The above results are obtained using the chemical potential of solid sulfur, a common approach for defect simulations in Sb_2_S_3_.^36,37,39^ To account for the volatility of sulfur at high temperatures, we also tested the effects of S vapor on defect concentrations (Section S2.5). While this change can decrease the absolute concentrations of *V*_S_, the concentrations remain insensitive to variations in chemical potential from Sb-rich to S-rich conditions. We note that postsulfurization steps in device fabrication are typically rapid and nonequilibrium, which may further influence defect populations and thus the photovoltaic conversion efficiency.

In conclusion, the low carrier concentrations and low *V*_OC_ in Sb_2_S_3_-based solar cells are linked to intrinsic point defects. The amphoteric nature of these defects leads to strong charge compensation and thus reduced carrier concentrations. The accessibility of multiple charge states for a single defect species is dealt with using Sah–Shockley statistics. Vacancies and antisites emerge as the most prevalent defects, with concentrations >10^12^ cm^–3^. Among these, sulfur vacancies are identified as the most detrimental, contributing substantially to the *V*_OC_ deficit. Our calculations show that band-to-band radiative and trap-mediated nonradiative recombination result in a *V*_OC_ loss up to 0.45 V, limiting the PCE to 16% under Sb-rich conditions. Notably, the equilibrium concentrations of key recombination centers are steadily high across various growth conditions due to defect-correlation effects, suggesting that their detrimental effects are inherent to the material and are challenging to mitigate. Therefore, effective defect engineering is crucial to improve the performance of Sb_2_S_3_ solar cells. While strategies to eliminate the harmful effects of sulfur vacancies remain an open question, studies suggest that oxygen or selenium may passivate these vacancies,^36,39^ though care must be taken to avoid the formation of secondary phases that could degrade performance.^7^ Postsulfurization treatments have been shown to enhance crystallinity and reduce recombination losses,^7,40^ and we find that S vapor can also reduce the equilibrium concentrations of S vacancies. Further research into optimized defect passivation techniques is necessary to unlock the full potential of Sb_2_S_3_-based solar cells.

## Methods

We predict the PCE of a single-junction solar cell by incorporating both radiative (band-to-band) and defect-mediated nonradiative recombination losses. The radiative limit is calculated using the bandgap, film thickness-dependent optical absorption, the standard AM1.5 solar spectrum, and an operating temperature of 300 K. This follows the methodology developed by Kim et al.^19,20^ The defect-mediated recombination rate is influenced by three primary factors: carrier capture coefficients, defect concentration, and recombination statistics.^41^ Here, we focus on the statistical modeling of recombination processes with other details provided in the Supporting Information.

The foundational theory of recombination via single-level defects was first established by Shockley, Read,^16^ and Hall.^17^ Sah and Shockley extended the statistics for defects with multiple energy levels.^24^ The key difference is that the Sah–Shockley theory accounts for correlated transitions between defects with different charge states, while the SRH model treats them as independent. The amphoteric model is reported to be necessary for systems with negative correlation energies^21^ such as antimony chalcogenides.^23^

**Figure 3:**
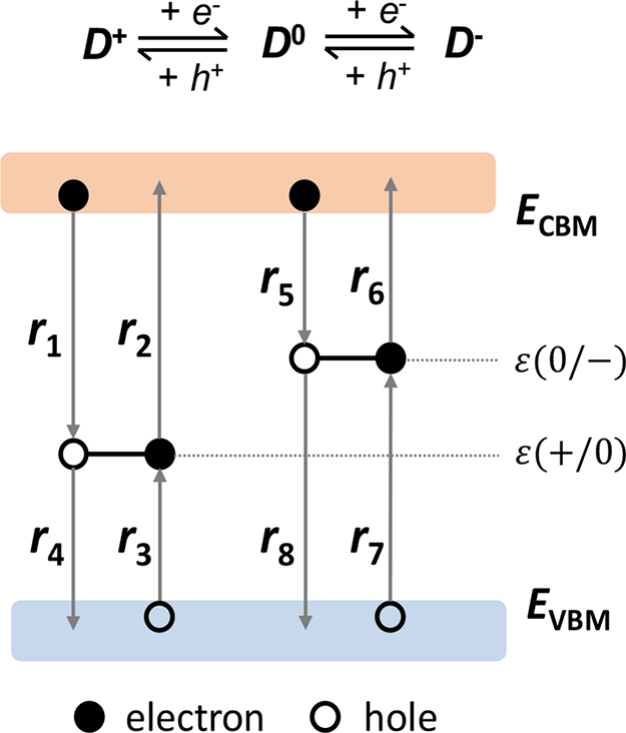
Schematic diagram of capture and emission processes for an amphoteric defect with two transition levels ε(+/0) and ε(0/−). Details of rates *r*_1_–*r*_8_ are provided in Table 2.

We illustrate the recombination statistics with an amphoteric defect that can exist in three charge states: *D*^+^, *D*^0^, and *D*^–^. The net recombination depends on eight individual (i.e., four capture and four emission) processes between these three states as shown in Figure 3. The corresponding capture and emission rates are listed in Table 2.

**Table 2:** Capture and Emission Processes in Amphoteric Defect^a^

Process	Transition	Rate
Electron capture *r*_1_	*D*^+^ + *e*^–^ → *D*^0^	*n C*_*n*_^+^ *N*_*T*_ *F*^+^
Electron emission *r*_2_	*D*^0^ → *D*^+^ + *e*^–^	*e*_*n*_^0^ *N*_*T*_ *F*^0^
Hole capture *r*_3_	*D*^0^ + *h*^+^ → *D*^+^	*p C*_*p*_^0^ *N*_*T*_ *F*^0^
Hole emission *r*_4_	*D*^+^ → *D*^0^ + *h*^+^	*e*_*p*_^+^ *N*_*T*_ *F*^+^
Electron capture *r*_5_	*D*^0^ + *e*^–^ → *D*^–^	*n C*_*n*_^0^ *N*_*T*_ *F*^0^
Electron emission *r*_6_	*D*^–^ → *D*^0^ + *e*^–^	*e*_*n*_^–^ *N*_*T*_ *F*^–^
Hole capture *r*_7_	*D*^–^ + *h*^+^ → *D*^0^	*p C*_*p*_^–^ *N*_*T*_ *F*^–^
Hole emission *r*_8_	*D*^0^ → *D*^–^ + *h*^+^	*e*_*p*_^0^ *N*_*T*_ *F*^0^

a *n* and *p* are the concentrations of electrons and holes, respectively. *C*_*n*/*p*_ and *e*_*n*/*p*_ are the capture and emission coefficients for electrons/holes, respectively. The superscript refers to the starting charge state of the process. *N*_T_ is the total concentration of defects. *F* is the occupation probability at a certain charge state.

Emission coefficients are derived from the principle of detailed balance^16^
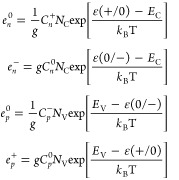
1where *N*_C_ and *N*_V_ are effective density of states in the conduction band (CB) and valence band (VB), respectively. *g* is the degeneracy factor, discussed in refs (42 and 43).

Under steady-state conditions, the net recombination is zero. By further considering the relation *F*^+^ + *F*^0^ + *F*^–^ = 1, the occupation functions are written as^44^
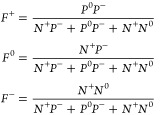
2where the variables *N*^+^, *N*^0^, *P*^–^, and *P*^0^ are defined as
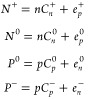
3

The net recombination rate *R* for an amphoteric defect is thus written as^44^
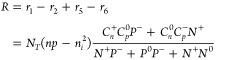
4where *N*_T_ is the total concentration of the defect with all possible charge states. *n* and *p* are concentrations of electrons and holes, respectively. *n*_i_ is the intrinsic carrier concentration. *C*_*n*/*p*_ is the capture coefficient for electrons/holes, and the superscript of capture coefficients refers to the starting charge state of the process. The total recombination rate is the sum of recombination rates for all defect species in a material.

## Supporting Information Available

Extended methods; defect thermodynamics; nonradiative carrier capture; simulated current–voltage curves (PDF)
