# Supplementary material for: Sulfur Vacancies Limit the Open-Circuit Voltage of Sb2S3 Solar Cells
Source: ACS Energy Lett. 2024 Dec 16;10(1):161–7. doi: 10.1021/acsenergylett.4c02722 (PMC11731329; doi:10.1021/acsenergylett.4c02722)
Supplement: Supplementary file 1 — nz4c02722_si_001.pdf [file nz4c02722_si_001.pdf]

Supporting Information
Sulfur Vacancies Limit the Open-circuit Voltage of Sb_2S_3 Solar Cells

Xinwei Wang,[†] Seán R. Kavanagh,[‡] and Aron Walsh[*,†,¶]

† *Department of Materials, Imperial College London, Exhibition Road, London SW7 2AZ, UK*

‡ *Center for the Environment, Harvard University, 29 Oxford St, Cambridge, MA 02138, US*

¶ *Department of Physics, Ewha Womans University, 52 Ewhayeodae-gil, Seodaemun-gu, Seoul, 03760, South Korea*

E-mail: a.walsh@imperial.ac.uk

S1. Computational methods

S1.1 Trap-limited conversion efficiency

By accounting for both radiative and non-radiative recombination processes, the net current density J under an applied bias voltage V can be expressed as

$$J(V;W) = J_{\mathrm{SC}}(W) + J_0^{\mathrm{rad}}(W)[1 - \exp(\frac{eV}{k_{\mathrm{B}}T})] - eR(V)W \tag{1}$$

where W is the film thickness. e is the elementary charge. J_0^{rad} is the saturation current. The short-circuit current J_{SC} is given by[1]

$$J_{\mathrm{SC}}(W) = e \int_{E_g}^{\infty} a(E; W)\Phi_{\mathrm{sun}}(E)\mathrm{d}E \tag{2}$$

where a is the photon absorptivity. $\Phi_{\mathrm{sun}}(E)$ is incident spectral photon flux density at the photon energy E.

The maximum efficiency is defined as the ratio of the maximum power density to the incident light power density, which is given by

$$\eta_{max} = \mathrm{max}_V \left(\frac{JV}{e\int_0^{\infty} E\Phi_{\mathrm{sun}}(E)\mathrm{d}E} \right) \tag{3}$$

S1.2 First-principles calculations

All electronic structure calculations were performed using Kohn-Sham density functional theory (DFT)[2,3] as implemented in Vienna Ab initio Simulation Package (VASP).[4] The projector augmented-wave (PAW) method[5] was employed with a converged plane-wave energy cutoff of 350 eV. Heyd-Scuseria-Ernzerhof hybrid exchange-correlation functional (HSE06)[6] and the D3 dispersion correction[7] were used for both geometry optimization and total energy calculations for each defect, which have been shown to accurately reproduce the structural and electronic properties of Sb_2S_3.[8] Spin-orbit coupling (SOC) effects have been reported to have a negligible impact on Sb_2S_3[9] and were thus not considered in this work.

Defect modeling. All point defects were simulated using a 3×1×1 supercell (with dimensions 11.39 Å×11.21 Å×11.39 Å) containing 60 atoms. This supercell size has been demonstrated to be adequate for defect calculations in this system,[10–12] given the strong screening effect to charged defects resulting from large dielectric constants of Sb_2S_3 (i.e. averaged static

dielectric constant $\varepsilon_0 = 68.76$).[8] The convergence criterion of forces on each atom was set to 0.01 eV/Å. For both geometry optimisation and static calculations, spin polarisation was turned on and a 2×2×2 Γ-centred k-point mesh was used. The ground-state defect configurations were obtained using the DOPED Python package (v0.0.7)[13] and ShakeNBreak.[14,15] The workflow of generating and optimizing defects follows the same procedures as described in our previous work.[16] The initial configurations for interstitial defects were generated using the Voronoi scheme.[17] This method identifies corners, edges and face centers of the Voronoi polyhedra as potential distinct sites, giving rise to twelve inequivalent interstitial sites in Sb_2S_3.

The formation energy of a point defect D in charge state q at a certain Fermi level E_F is calculated by[18,19]

$$\Delta E^f_{D,q} = E_{D,q} - E_{\text{host}} - \sum_i n_i\mu_i + qE_F + E_{\text{corr}} \tag{4}$$

where $E_{D,q}$ and E_{host} are the total energies of the supercell with the defect D and the pristine supercell, respectively. $\sum_i n_i\mu_i$ accounts for the energy cost of adding ($n_i > 0$) or removing ($n_i < 0$) n_i atoms of type i with their reservoir chemical potential μ_i. Details about the chemical potential limits are shown in Section S2.1. E_{corr} accounts for the finite-size corrections for charged defects under periodic boundary conditions. To accurately account for anisotropic dielectric screening in defect formation energy calculations, the correction scheme developed by Kumagai and Oba[20] is employed. Correction energies of sulfur vacancies are shown as examples in Table S1.

The thermodynamic charge transition level (TL) $\varepsilon(q_1/q_2)$ is defined as the Fermi-level posi-

tion at which a defect with q_1 and q_2 charge states have the same formation energies

$$\varepsilon(q_1/q_2) = \frac{\Delta E^f_{D,q_1}(E_F = 0) - \Delta E^f_{D,q_2}(E_F = 0)}{q_2 - q_1} \tag{5}$$

where $\Delta E^f_{D,q}(E_F = 0)$ is the formation energy of a defect D in the charge state q when the Fermi level is at the valence band maximum (VBM). The TL diagram of Sb_2S_3 is shown in Fig. S3.

The equilibrium concentration of defects, $C_{D,q}$, is given as

$$C_{D,q} = g_{\mathrm{Total}} N_D e^{\frac{-\Delta E^f_{D,q}}{k_{\mathrm{B}} T_{\mathrm{anneal}}}} \tag{6}$$

where g_{Total} is the total degeneracy factor, including geometry (g_{Geo}) and spin (g_{Spin}) contributions. As an example, sulfur vacancy degeneracies are listed in Table S1. Here, N_D is the number of possible defect sites per unit volume in the supercell. k_{B} is the Boltzmann constant, and T_{anneal} is the temperature at which the host material is annealed/synthesized. $\Delta E^f_{D,q}$ represents the formation energy of defect D,q at the self-consistent Fermi level.

The self-consistent Fermi level E_F is determined by the density of all charged defects and calculated iteratively based on the charge neutrality condition[21,22]

$$\sum_{D,q} qC_{D,q} - n_0 + p_0 = 0 \tag{7}$$

where $C_{D,q}$ is the concentration of defect D in its accessible charge state q. n_0 and p_0 are the concentrations of free electrons and holes, respectively.

We note that many experimental processes, such as the rapid quenching, often occur far from equilibrium.[23] Under such conditions, defects can become 'frozen in' at the annealing

temperature due to kinetic barriers. In our simulations, defect concentrations are calculated at T_{anneal}, corresponding to the self-consistent Fermi level denoted as annealing E_F). During quenching, the total concentration of each defect type remains fixed, while changes in defect charge states and free carrier concentrations are allowed to reach charge neutrality at the measurement temperature T, with the corresponding self-consistent Fermi level denoted as quenched E_F). Therefore, in defect formation energy diagrams, formation energies of defects are obtained using the annealing E_F, whereas their charge states are determined using the quenched E_F.

S2. Defect thermodynamics in Sb_2S_3

S2.1 Chemical potential limits

Since Sb_2S_3 has no stable non-elemental competing phases, the boundaries of the chemical potential ranges of μ_{Sb} and μ_{S} are determined by the following restrictions:
i) the sum of atomic chemical potentials should be at equilibrium with the formation of Sb_2S_3

$$2\Delta\mu_{\mathrm{Sb}} + 3\Delta\mu_{\mathrm{S}} = \Delta H_{\mathrm{f}}(\mathrm{Sb_2S_3}) \tag{8}$$

where $\Delta\mu_{\mathrm{Sb/S}}$ is referenced to the standard elemental phases. $\Delta H_{\mathrm{f}}(\mathrm{Sb_2S_3})$ is the formation energy of Sb_2S_3.
ii) the formation of elemental phases of Sb and S should be avoided

$$\begin{aligned} \Delta\mu_{\mathrm{Sb}} &\leq 0 \\ \Delta\mu_{\mathrm{S}} &\leq 0 \end{aligned} \tag{9}$$

Calculated chemical potential limits are shown in Fig. S1. Initial competing phases are generated using DOPED,[13] taking all Materials Project phases which could border the host material chemical stability region with an error bar of 0.07 eV/atom, before recom-

puting with the HSE06 hybrid functional[6] and D3 dispersion correction.[7] Under Sb-rich conditions, $\Delta\mu_{\mathrm{Sb}}$=0 eV and $\Delta\mu_{\mathrm{S}}$=−0.42 eV. While under S-rich conditions, $\Delta\mu_{\mathrm{S}}$=0 eV and $\Delta\mu_{\mathrm{Sb}}$=−0.62 eV. Various growth conditions are obtained by linearly interpolating these two extreme chemical potentials.

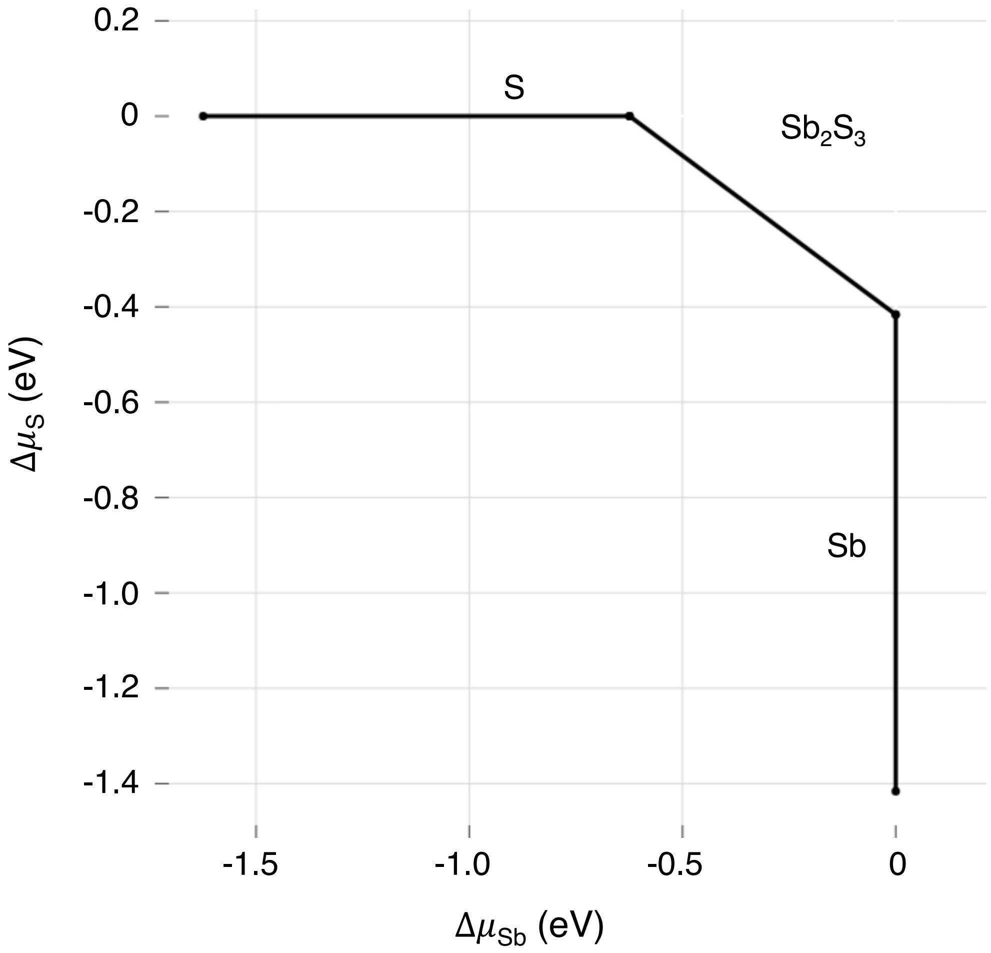

Figure S1: Calculated chemical potential limits of Sb_2S_3. $\Delta\mu_{\mathrm{Sb/S}}$ is referenced to the standard elemental phases.

S2.2 Defect formation energy diagrams under various growth conditions

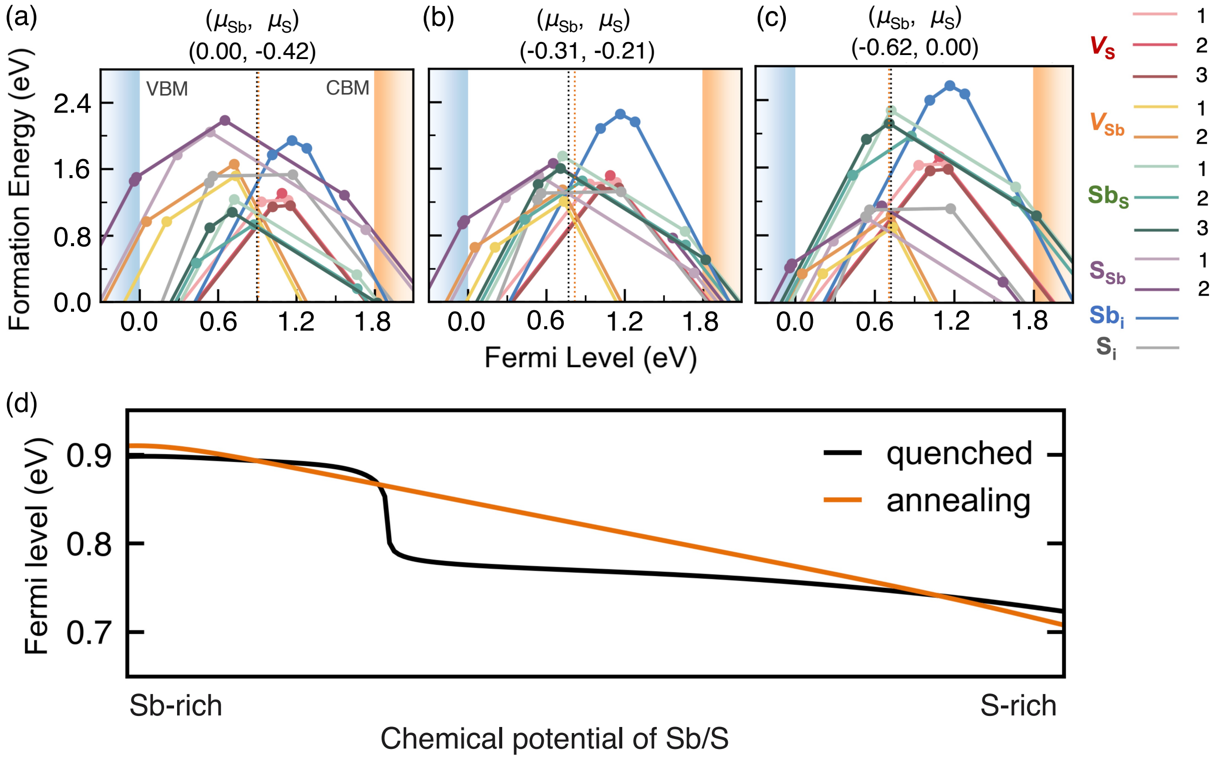

Figure S2: (a-c) Formation energies of all intrinsic point defects in Sb_2S_3 under (a) Sb-rich, (b) Sb-moderate and (c) S-rich conditions. Vertical dashed lines in orange and black, respectively, indicate self-consistent Fermi levels at 603 K [24,25] and at 300 K in Sb_2S_3 crystals grown at 603 K.[24,25] (d) Self-consistent Fermi level (relative to the VBM) as a function of the growth condition.

The formation energies of defects are determined by the self-consistent Fermi level (E_F) and different elemental chemical potentials. Fig. S2(a)-(c) show the calculated defect formation energy diagrams under Sb-rich, Sb-moderate, and Sb-poor conditions, respectively. $V_{S(2)}$, $V_{S(3)}$ and $V_{Sb(1)}$ have relatively low formation energies under various growth conditions, which are consistent with their high concentrations shown in Fig. 1(c). Under Sb-rich conditions, $Sb_{S(2)}$ and $Sb_{S(3)}$ are the lowest-energy defects; while under S-rich conditions, $S_{Sb(1)}$ has the lowest energy.

All intrinsic point defects in Sb_2S_3 are amphoteric with both positive and negative charge

states. The strong charge compensation from the low-energy defects thus pins the self-consistent E_F close to the middle of the band gap (Fig. S2(d)), which agrees well with experimental observations.[26] With the increase of μ_S, the quenched (annealing) self-consistent E_F decreases from 0.90 (0.91 eV) under Sb-rich conditions to 0.72 (0.71 eV) under S-rich conditions. Considering the calculated fundamental band gap of 1.79 eV, this indicates intrinsic weakly p-type conductivity.

S2.3 Transition energy levels of defects

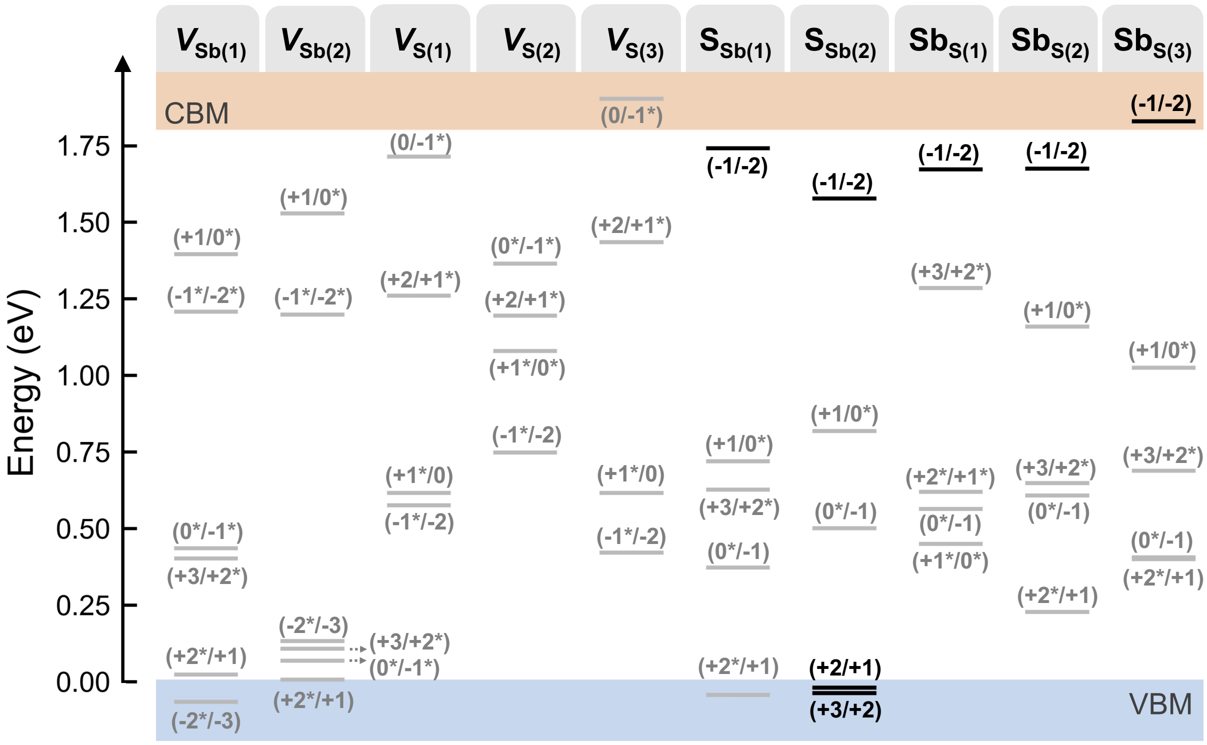

Figure S3: Calculated charge state transition levels (TLs) of intrinsic point defects with high concentrations in Sb_2S_3 using Equation S5. Metastable charge states are indicated with asterisks (*), and TLs with metastable charge states are shown in grey. The Fermi level is referenced to the valence band maximum (VBM).

S2.4 Correction energies and degeneracy factors for sulfur vacancies in Sb_2S_3

Table S1: Correction energies in Equation S4 and degeneracy factors in Equation S6 of sulfur vacancies in Sb_2S_3

Defect	Charge state	g_{Geo}	g_{Spin}	g_{Total}	E_{corr}
	+2	2	1	2	-0.25
	+1	1	2	2	-0.08
$V_{\text{S(1)}}$	0	1	1	1	0.00
	-1	2	2	4	0.16
	-2	2	1	2	0.31
	+2	1	1	1	-0.26
	+1	2	2	4	-0.14
$V_{\text{S(2)}}$	0	2	1	2	0.00
	-1	2	2	4	0.12
	-2	2	1	2	0.26
	+2	1	1	1	-0.26
	+1	1	2	2	-0.14
$V_{\text{S(3)}}$	0	2	1	2	0.00
	-1	1	2	2	0.12
	-2	2	1	2	0.24

S2.5 Defect thermodynamics in equilibrium with sulfur vapor

The results shown in the main text and Sections S2.1-S2.4 were obtained using the chemical potential of solid sulfur, a common approach in defect simulations for Sb_2S_3.[10,12,27] However, sulfur is highly volatile and can transition into the gas phase at high temperatures. To account for this, we also examined the impact of using the chemical potential of sulfur vapor on our conclusions. A parametrized model[28] for sulfur vapor was applied to incorporate the effects of sulfur partial pressure in both the S_2 and S_8 allotropes. We assumed an annealing temperature of 603 K for Sb_2S_3 samples under a pressure of 1×10^4 Pa.

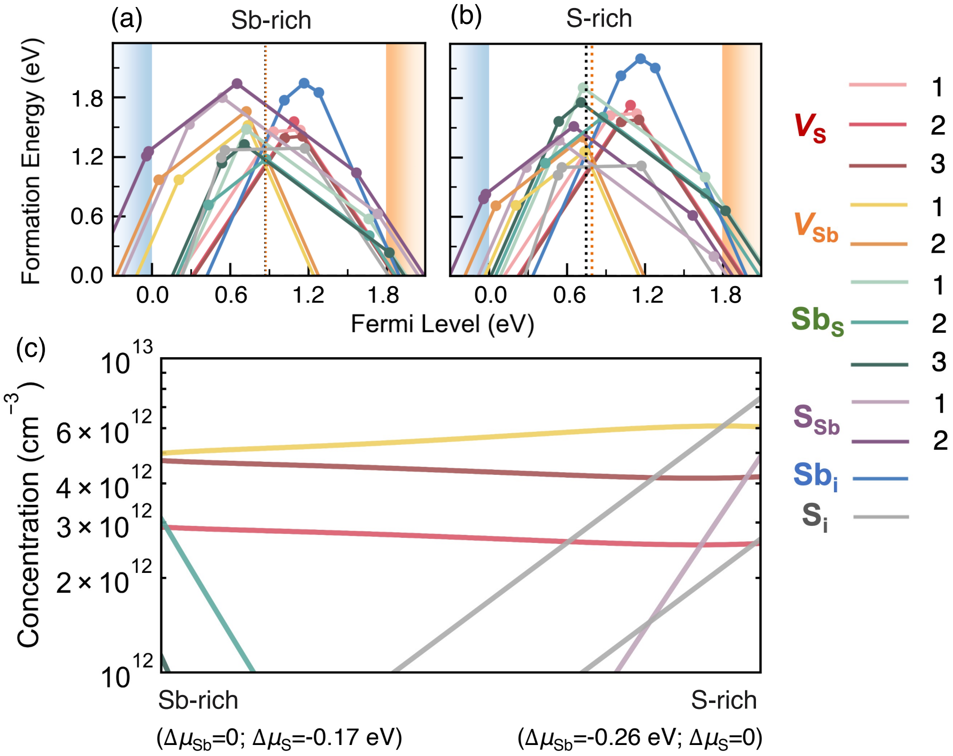

Figure S4: (a-b) Calculated formation energies of intrinsic point defects in Sb_2S_3 under (a) Sb-rich and (b) S-rich growth conditions using the chemical potential of sulfur vapor at 603 K and 1×10^4 Pa. The orange and black dashed lines indicate self-consistent Fermi levels at 603 K[24,25] and at 300 K in Sb_2S_3 crystals grown at 603 K,[24,25] respectively. (b) Equilibrium defect concentration at 300 K in Sb_2S_3 crystals grown at 603 K[24,25] as a function of growth condition.

As shown in Fig. S4, the equilibrium concentrations of $V_{S(2)}$ and $V_{S(3)}$ decrease by over an order of magnitude due to increased formation energies, suggesting a reduced impact on performance. However, their concentrations remain relatively insensitive to variations in chemical potential. These findings suggest that modifying the chemical environment alone is unlikely to enhance Sb_2S_3 performance.

S3. Non-radiative carrier capture processes

The non-radiative carrier capture pathways involving intrinsic point defects in Sb_2S_3 are shown in Fig. S5. The carrier capture processes are investigated following the workflow illustrated in Fig. S6:

i) For each defect D, we start with its thermodynamically stable charge state q at the calculated self-consistent Fermi level, denoted as D^q (bolded in Fig. S5). If D^q has a low equilibrium concentration under various growth conditions, transitions involving this defect are ruled out. Specifically, $V_{S(1)}$ and all interstitials in Sb_2S_3 are excluded due to their low concentrations.

ii) For defects with high concentrations, thermodynamic charge transition levels (TLs) are calculated using Equation S5. When a defect level lies very close to the band edges, capture and emission of carriers of the same type are both rapid (denoted by a rocket symbol in Fig. S5), which precludes the defect from acting as an effective recombination center. In this study, we exclude transitions where the defect level lies within 50 meV of the band edges, approximately twice the thermal energy at room temperature. Examples of these disregarded transitions include those between between Sb_S^- and Sb_S^{2-} for all inequivalent sites.

iii) For defect states passing conditions i) and ii), the electron and hole capture coefficients are then calculated for transitions between D^q and D^{q+1}/D^{q-1}. The relative defect concentrations under steady-state conditions are then recalculated using Equations 1-3 shown in Method. If D^{q+1}/D^{q-1} has an extremely low steady-state concentration (below 10^{-25} cm^3/s), adjacent transitions are excluded (labeled as '∅' in Figure S5). Otherwise, we further consider neighboring transitions of $D^{q+1} \leftrightarrow D^{q+2}$ / $D^{q-1} \leftrightarrow D^{q-2}$. During this step, if a large mass-weighted displacement ΔQ indicates significant structural dissimilarity between two states, that transition is excluded (marked by an 'X' in Fig. S5). In such cases, metastable defect structures are considered as potential intermediate configurations in the capture processes.[29,30] Examples of such exclusions include transitions between $S_{Sb(2)}^-$ and $S_{Sb(2)}^0$.

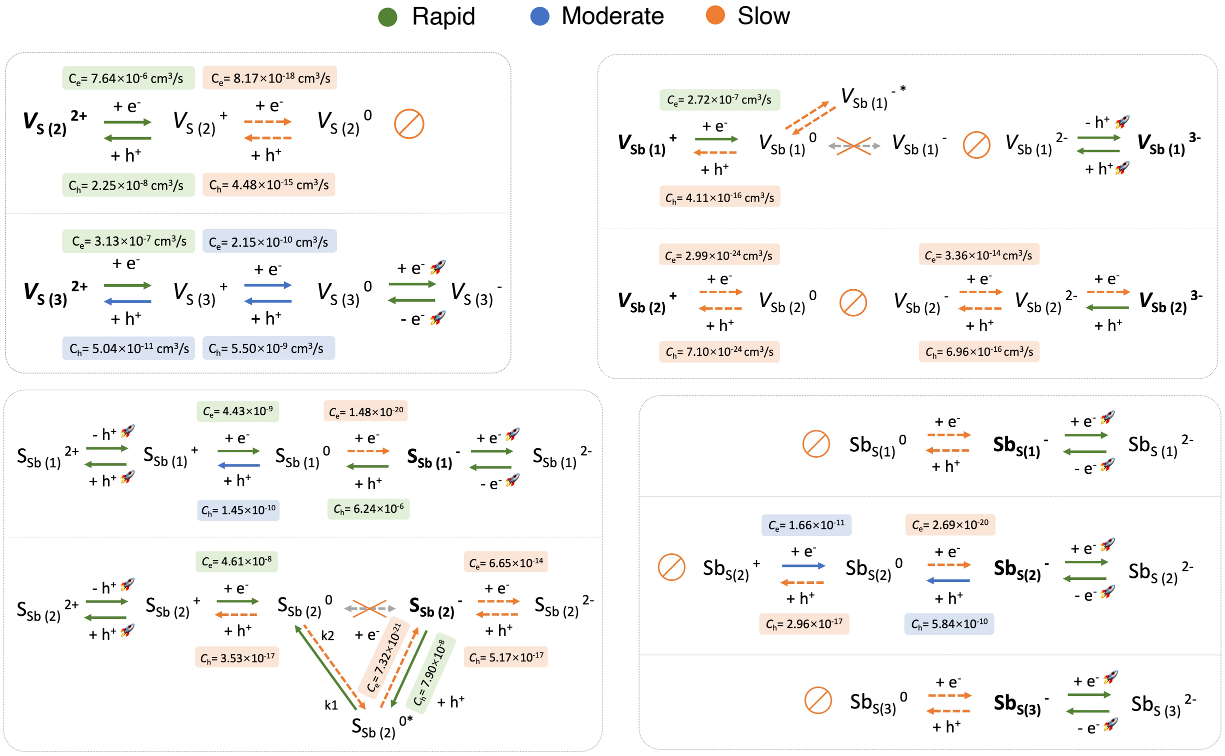

Figure S5: Pathways for electron and hole capture via traps with high carrier concentrations in Sb_2S_3. Defect species in bold are thermodynamically stable states at calculated self-consistent Fermi levels, which are the most likely starting points in capture processes. The defect species with superscript asterisks refer to metastable defect configurations. C_e and C_h are electron and hole capture coefficients, respectively. Green, blue and orange colours indicate rapid, intermediate and slow capture. '⊘' refers to transitions from states with extremely low predicted concentrations under illumination. Transitions with large mass-weighted displacements are also ruled out, as indicated by an 'X' mark. Capture coefficients smaller than 10^{-25} cm^3/s are not shown.

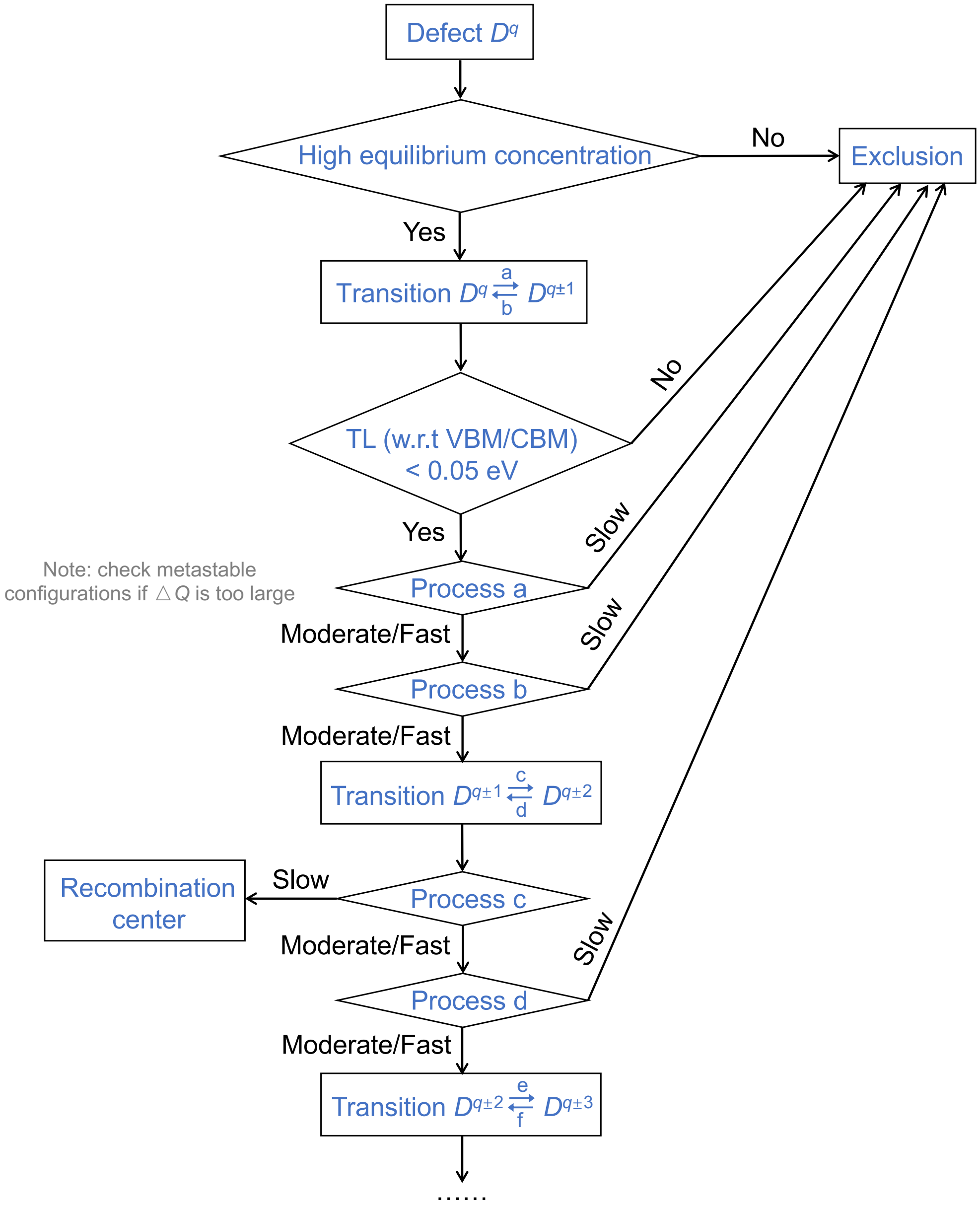

Figure S6: Schematic diagram of the workflow used to investigate carrier capture processes in Fig. S5.

S4. Trap-limited conversion efficiency

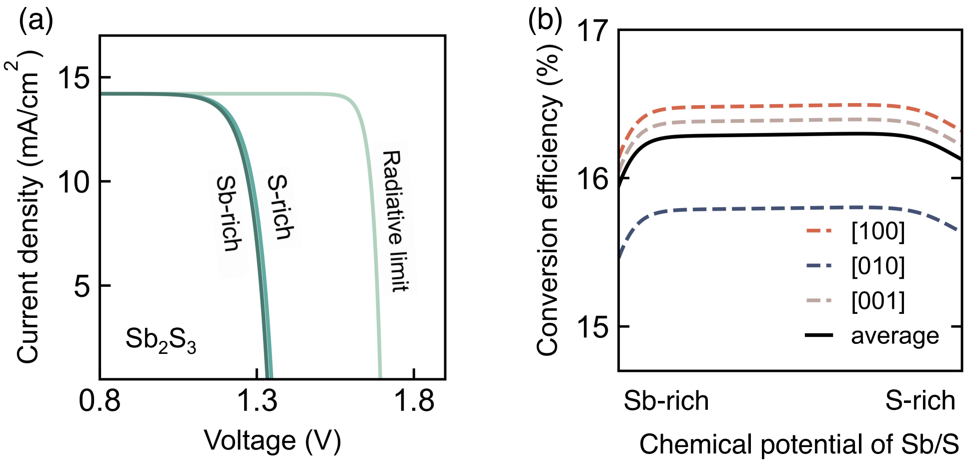

Figure S7: (a) Calculated current density-voltage ($J-V$) curves for Sb_2S_3, assuming the radiative limit (only band-to-band radiative recombination losses) and including defect-induced non-radiative recombination under S/Sb-rich growth conditions. The radiative limit is calculated by averaging the optical absorption coefficients along [100], [010] and [001] crystallographic directions. (b) Trap-limited conversion efficiency as a function of the growth condition. [100], [010] and [001] correspond to the crystallographic directions in Sb_2S_3. All results shown correspond to a film thickness of 400 nm, and room temperature defect concentrations assuming an annealing temperature of 603 K.[24,25]

Fig. S7(a) shows the predicted current density-voltage (J-V) curves for Sb_2S_3 solar cells, incorporating both the radiative limit and trap-assisted non-radiative recombination processes. The radiative limit is predicted based on the calculated band gap, directionally-averaged optical absorption coefficients, and an assumed film thickness of 400 nm.[24] The predicted short-circuit current density (J_{SC}) is 14.2 mA/cm^2 (Fig. S7(a)), which is lower than the J_{SC} of 19.3 mA/cm^2 observed in the highest-efficiency Sb_2S_3 solar cell.[24] This discrepancy is attributed to the exclusion of temperature effects in the model, which would otherwise increase the calculated J_{SC}. Additionally, the predicted open-circuit voltage V_{OC} deficit due to radiative recombination is 0.10 V. Non-radiative recombination significantly contributes to V_{OC} deficit, with total predicted deficits of 0.45 V and 0.44 V under Sb-rich and S-rich conditions, respectively (Fig. S7(a)).

The upper limit to conversion efficiencies in Sb_2S_3 solar cells have been predicted using the TLC model[1,31] as shown in Fig. S7(b). The anisotropic conversion efficiency was cal-

culated based on the corresponding optical absorption coefficients. Our predictions indicate that the highest trap-limited conversion efficiency of 16.5 % can be achieved along the [100] direction, which corresponds to the direction parallel to the quasi-one-dimensional $[Sb_4S_6]_n$ ribbons under optimal growth conditions in Sb_2S_3. However, under the same conditions, the maximum difference in efficiency along different directions is only 0.69 %.

S5. Electron-phonon matrix element calculations

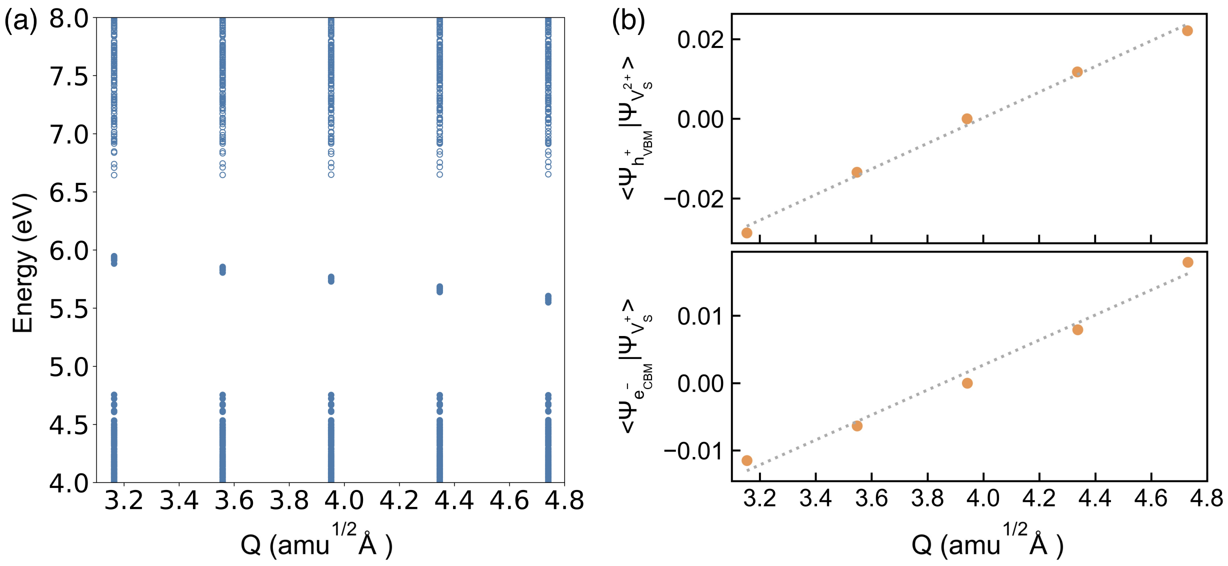

Figure S8: Calculation of the electron-phonon matrix element for $V_{\mathrm{S(2)}}^{2+} \leftrightarrow V_{\mathrm{S(2)}}^{+}$ transition. (a) Eigenvalues (spin-up channel) of defect states and band edge wavefunctions as a function of the generalized coordinate Q. (b) Overlap integral $\langle \psi_i | \psi_f \rangle$ as a function of Q for hole and electron capture processes.

The carrier capture coefficients are calculated using the electron-phonon coupling matrix element, which is expanded in a Taylor series around Q_0, under the linear-coupling approximation.[32] Fig. S8 presents the datapoints used to calculate the electron-phonon matrix element for the $V_{\mathrm{S(2)}}^{2+} \leftrightarrow V_{\mathrm{S(2)}}^{+}$ transition. In Fig. S8(a), the eigenvalues of the spin-up channel defect state exhibit a linear dependence on the generalized coordinate Q, while the valence band maximum (VBM) and conduction band minimum (CBM) remain constant with

respect to Q. Fig. S8(b) illustrates the overlap integral $\langle \psi_i | \psi_f \rangle$ as a function of Q for both hole and electron capture processes, with linear fits applied to each.

References

(1) Kim, S.; Márquez, J. A.; Unold, T.; Walsh, A. Upper limit to the photovoltaic efficiency of imperfect crystals from first principles. *Energy Environ. Sci.* **2020**, *13*, 1481–1491.

(2) Kohn, W.; Sham, L. J. Self-consistent equations including exchange and correlation effects. *Phys. Rev.* **1965**, *140*, A1133.

(3) Dreizler, R. M.; Gross, E. K. *Density Functional Theory*; Springer: Berlin, Heidelberg, 1990; pp 245–271.

(4) Kresse, G.; Furthmüller, J. Efficient iterative schemes for ab initio total-energy calculations using a plane-wave basis set. *Phys. Rev. B* **1996**, *54*, 11169.

(5) Kresse, G.; Joubert, D. From ultrasoft pseudopotentials to the projector augmented-wave method. *Phys. Rev. B* **1999**, *59*, 1758.

(6) Heyd, J.; Scuseria, G. E.; Ernzerhof, M. Hybrid functionals based on a screened Coulomb potential. *J. Chem. Phys.* **2003**, *118*, 8207–8215.

(7) Grimme, S. Accurate description of van der Waals complexes by density functional theory including empirical corrections. *J. Comput. Chem.* **2004**, *25*, 1463–1473.

(8) Wang, X.; Li, Z.; Kavanagh, S. R.; Ganose, A. M.; Walsh, A. Lone pair driven anisotropy in antimony chalcogenide semiconductors. *Phys. Chem. Chem. Phys.* **2022**, *24*, 7195–7202.

(9) Filip, M. R.; Patrick, C. E.; Giustino, F. GW quasiparticle band structures of stibnite, antimonselite, bismuthinite, and guanajuatite. *Phys. Rev. B* **2013**, *87*, 205125.

(10) Zhao, R.; Yang, X.; Shi, H.; Du, M.-H. Intrinsic and complex defect engineering of quasi-one-dimensional ribbons Sb_2S_3 for photovoltaics performance. *Phys. Rev. Mater.* **2021**, *5*, 054605.

(11) Chen, S.; Li, M.; Zhu, Y.; Cai, X.; Xiao, F.; Ma, T.; Yang, J.; Shen, G.; Ke, A.; Lu, Y.; others A codoping strategy for efficient planar heterojunction Sb_2S_3 solar cells. *Adv. Energy Mater.* **2022**, *12*, 2202897.

(12) Zhang, Z. Sulfur-Vacancy Passivation via Selenium Doping in Sb_2S_3 Solar Cells: Density Functional Theory Analysis. *J. Phys. Chem. C* **2022**, *126*, 20786–20792.

(13) Kavanagh, S. R.; Squires, A. G.; Nicolson, A.; Mosquera-Lois, I.; Ganose, A. M.; Zhu, B.; Brlec, K.; Walsh, A.; Scanlon, D. O. doped: Python toolkit for robust and repeatable charged defect supercell calculations. *J. Open Source Softw.* **2024**, *9*, 6433.

(14) Mosquera-Lois, I.; Kavanagh, S. R.; Walsh, A.; Scanlon, D. O. ShakeNBreak: Navigating the defect configurational landscape. *J. Open Source Softw.* **2022**, *7*, 4817.

(15) Mosquera-Lois, I.; Kavanagh, S. R.; Walsh, A.; Scanlon, D. O. Identifying the ground state structures of point defects in solids. *npj Comput. Mater.* **2023**, *9*, 25.

(16) Wang, X.; Kavanagh, S. R.; Scanlon, D. O.; Walsh, A. Upper efficiency limit of Sb_2Se_3 solar cells. *Joule* **2024**, *8*, 2105–2122.

(17) Rycroft, C. VORO++: A three-dimensional Voronoi cell library in C++. **2009**,

(18) Zhang, S.; Northrup, J. E. Chemical potential dependence of defect formation energies in GaAs: Application to Ga self-diffusion. *Phys. Rev. Lett.* **1991**, *67*, 2339.

(19) Freysoldt, C.; Grabowski, B.; Hickel, T.; Neugebauer, J.; Kresse, G.; Janotti, A.; Van de Walle, C. G. First-principles calculations for point defects in solids. *Rev. Mod. Phys.* **2014**, *86*, 253.

(20) Kumagai, Y.; Oba, F. Electrostatics-based finite-size corrections for first-principles point defect calculations. *Phys. Rev. B.* **2014**, *89*, 195205.

(21) Squires, A. G.; Scanlon, D. O.; Morgan, B. J. py-sc-fermi: self-consistent Fermi energies and defect concentrations from electronic structure calculations. *J. Open Source Softw.* **2023**, *8*.

(22) Buckeridge, J. Equilibrium point defect and charge carrier concentrations in a material determined through calculation of the self-consistent Fermi energy. *Comput. Phys. Commun.* **2019**, *244*, 329–342.

(23) Bachrach, R.; Krusor, B. Morphological defects arising during MBE growth of GaAs. *J. Vac. Sci. Technol.* **1981**, *18*, 756–764.

(24) Zhu, L.; Liu, R.; Wan, Z.; Cao, W.; Dong, C.; Wang, Y.; Chen, C.; Chen, J.; Naveed, F.; Kuang, J.; others Parallel Planar Heterojunction Strategy Enables Sb_2S_3 Solar Cells with Efficiency Exceeding 8%. *Angew. Chem.* **2023**, *135*, e202312951.

(25) Chen, J.; Qi, J.; Liu, R.; Zhu, X.; Wan, Z.; Zhao, Q.; Tao, S.; Dong, C.; Ashebir, G. Y.; Chen, W.; others Preferentially oriented large antimony trisulfide single-crystalline cuboids grown on polycrystalline titania film for solar cells. *Commun. Chem.* **2019**, *2*, 121.

(26) Savadogo, O.; Mandal, K. Low cost Schottky barrier solar cells fabricated on CdSe and Sb_2S_3 films chemically deposited with silicotungstic acid. *J. Electrochem. Soc.* **1994**, *141*, 2871.

(27) Cai, Z.; Dai, C.-M.; Chen, S. Intrinsic defect limit to the electrical conductivity and a two-step p-type doping strategy for overcoming the efficiency bottleneck of Sb_2S_3-based solar cells. *Sol. RRL* **2020**, *4*, 1900503.

(28) Jackson, A. J.; Tiana, D.; Walsh, A. A universal chemical potential for sulfur vapours. *Chem. Sci.* **2016**, *7*, 1082–1092.

(29) Kavanagh, S. R.; Scanlon, D. O.; Walsh, A.; Freysoldt, C. Impact of metastable defect structures on carrier recombination in solar cells. *Faraday Discuss.* **2022**, *239*, 339–356.

(30) Alkauskas, A.; Dreyer, C. E.; Lyons, J. L.; Van de Walle, C. G. Role of excited states in Shockley-Read-Hall recombination in wide-band-gap semiconductors. *Phys. Rev. B* **2016**, *93*, 201304.

(31) Kim, S.; Walsh, A. Ab initio calculation of the detailed balance limit to the photovoltaic efficiency of single pn junction kesterite solar cells. *Appl. Phys. Lett.* **2021**, *118*.

(32) Alkauskas, A.; Yan, Q.; Van de Walle, C. G. First-principles theory of nonradiative carrier capture via multiphonon emission. *Phys. Rev. B* **2014**, *90*, 075202.
